# 604. IL-17C as an early diagnostic biomarker for invasive fungal infection or just a marker of any infection in at-risk haematology patients

**DOI:** 10.1093/ofid/ofad500.671

**Published:** 2023-11-27

**Authors:** Robina Aerts, Isis Ricano Ponce, Mariolina Bruno, Toine Mercier, Diletta Rosati, Johan A Maertens, Vinod Kumar, Agostinho Carvalho, Mihai Netea, Martin Hoenigl

**Affiliations:** University Hospitals Leuven, Leuven, Limburg, Belgium; Radboud UMC, Nijmegen, Gelderland, Netherlands; Radboud UMC, Nijmegen, Gelderland, Netherlands; University Hospitals Leuven, Leuven, Limburg, Belgium; Radboud UMC, Nijmegen, Gelderland, Netherlands; KU Leuven, Leuven, Brussels Hoofdstedelijk Gewest, Belgium; Radboud UMC, Nijmegen, Gelderland, Netherlands; UMinho, Braga, Braga, Portugal; Radboud University Medical Center, Nijmegen, Netherlands; Medical University of Graz, Department of Internal Medicine, Division of Infectious Diseases, Graz, Steiermark, Austria

## Abstract

**Background:**

As mortality of invasive mould infections (IMI) remains high, there is a need for improved biomarkers for timely diagnosis and patient stratification. Various moulds have been shown to induce T-helper cell (Th) 1, Th2, Th9 and Th17 subsets resulting not in potent anti-Aspergillus T-cells effector mechanisms, and in elevated serum levels of cytokines such as IFN-γ and IL-6, IL-8, IL-15 and IL-17. The ECMM study “Immunologic Markers for Treatment Monitoring and Diagnosis in Invasive Mold Infection” aimed to identify circulating immunological markers that could be useful for an early diagnosis of IMI.

**Methods:**

We collected longitudinal serum samples from 33 cases with probable/proven IMI and two matched control cohorts without IMI, and from an independent validation cohort with 20 cases and 20 matched controls (Figure 1, Table 1). None of the patients received mold active prophylaxis. A panel of 92 circulating proteins involved in inflammation was measured using a targeted proteomics platform (Olink Proteomics AB (Uppsala, Sweden)) and protein concentrations were compared. Correction for multiple testing was performed by Benjamini-Hochberg method.

Figure 1

**Figure:**
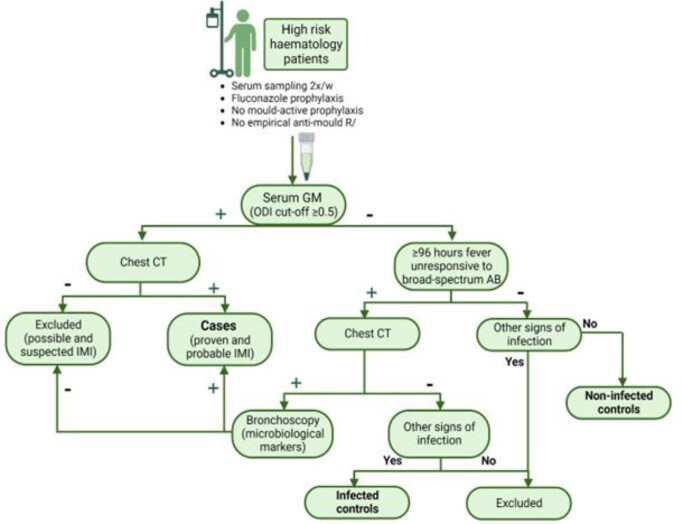

Overview of sample collection

Table 1

**Figure:**
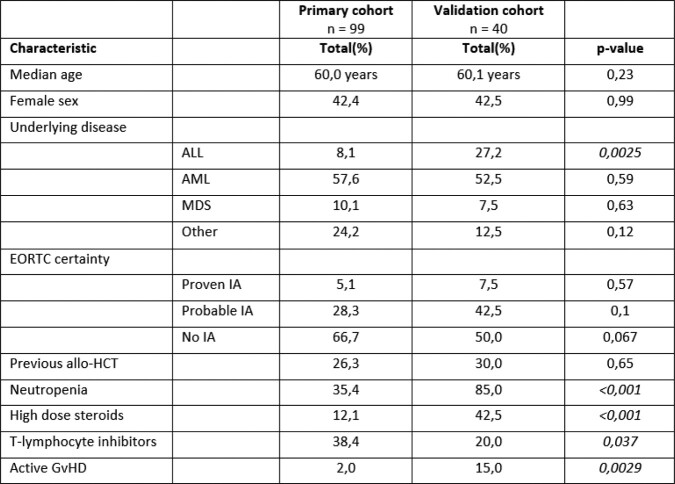

An overview of the main characteristics of and differences between the derivation cohort and the validation cohort, including characteristics of all cases and all controls.

**Results:**

Abundance analysis on the day of diagnosis revealed 30 differentially regulated proteins. Nine were replicated in an independent cohort: MMP-10, IL-18, IL-18-R1, IL10, CDCP1 and IL-17C. In the analysis of serum samples collected more than 10 days before diagnosis, six proteins showed higher concentrations in the cases compared to the controls (CCL20, IL-6, IL-17C, CCL23, CXCL5, CXCL1 and CX3CL1), while nine proteins had lower concentrations (Figure 2). The higher IL-17C concentration could be replicated in the independent validation cohort (Figure 3). When comparing cases only to infected controls this difference in expression was not significant anymore after correcting for multiple testing.

Figure 2

**Figure:**
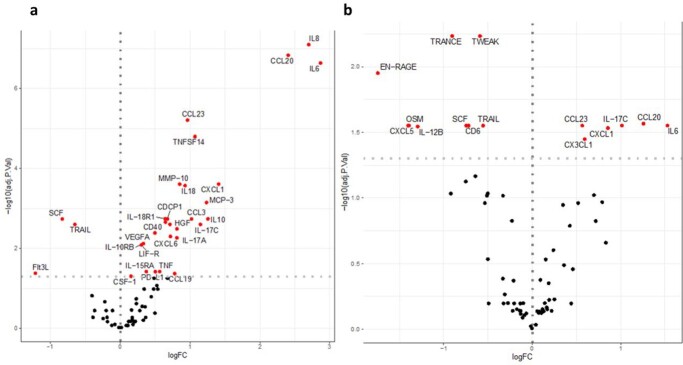

Figure 3

**Figure:**
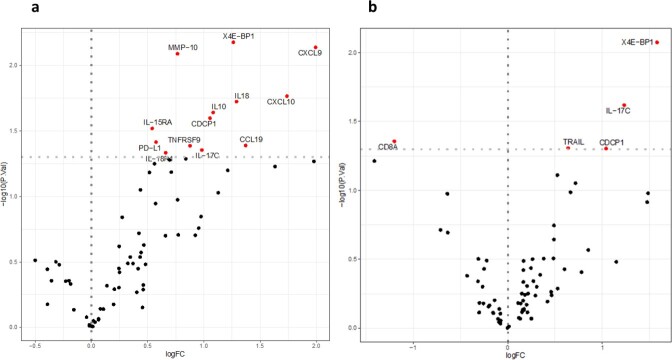

**Conclusion:**

We found a consistent higher expression of IL-17C in cases with IMI versus non-infected controls, both at the time of diagnosis and in samples 10 days before the diagnosis of IMI. IL-17C, known for its pro-inflammatory function at the epithelial sites, activates expression of other proinflammatory cytokines and of chemokines such as CXCL1/2/3 and CCL20, and could present an early biomarker for IMI.

Abundance analysis of the primary cohort (a) at time of diagnosis, and (b) at more than 10 days before diagnosis, comparing cases with non infected controls.

Abundance analysis of the validation cohort (a) at time of diagnosis, and (b) at more than 10 days before diagnosis, comparing cases with non infected controls.

Table 2-1

Table 2

Clinical and microbiological information of the 33 cases included in the primary cohort and the 20 cases included in the validation cohort PART 1.

**Disclosures:**

**Martin Hoenigl, n/a**, Astellas: Grant/Research Support|Euroimmun: Grant/Research Support|F2G: Grant/Research Support|Gilead: Grant/Research Support|Immy: Grant/Research Support|MSD: Grant/Research Support|Mundipharma: Grant/Research Support|Pfizer: Grant/Research Support|Pulmocide: Grant/Research Support|Scynexis: Grant/Research Support

**Table:** 

Primary cohort										
Case	Sex	Age	Underlying disease	EORTC certainty	Treatment	Previous allo-HCT	Neutro-penia	High dose steroids	T-lymphocyte inhibitors	EORTC response week 6
1	F	45	MDS	Probable	Caspofungin	Yes	Yes	No	Yes	Death
2	M	53	AML	Probable	Posaconazole VS voriconazole	Yes	Yes	No	Yes	Complete
3	M	68	AML	Probable	Posaconazole VS voriconazole	No	No	No	Yes	Complete
4	M	54	AML	Probable	Caspofungin	No	Yes	No	Yes	Partial
5	M	37	ALL	Probable	Voriconazole	Yes	No	No	No	Death
6	M	58	AML	Proven	Voriconazole	No	No	No	Yes	Partial
7	F	35	MF	Proven	AmphotericinB	No	Yes	No	Yes	Complete
8	F	81	AML	Proven	Voriconazole	No	No	No	No	Partial
9	M	37	Lymphoma	Probable	Voriconazole	Yes	No	No	No	Partial
10	F	54	AML	Probable	AmphotericinB	No	No	No	Yes	Partial
11	M	34	Lymphoma	Probable	Voriconazole	Yes	No	No	Yes	Partial
12	M	44	Lymphoma	Proven	Posaconazole	Yes	Yes	Yes	No	Death
13	M	56	AML	Probable	Voriconazole	No	No	No	Yes	Complete
14	M	61	ALL	Probable	Posaconazole	No	Yes	No	Yes	Progression
15	M	71	AML	Probable	AmphotericinB	No	Yes	No	Yes	Death
16	M	68	AML	Probable	Caspofungin	No	Yes	No	Yes	Death
17	F	54	AML	Probable	Olorofim	Yes	No	No	Yes	Death
18	F	49	AML	Probable	Voriconazole	Yes	Yes	No	No	Death
19	M	67	AML	Probable	Voriconazole	No	Yes	No	Yes	Partial
20	F	28	Lymphoma	Probable	AmphotericinB	No	No	Yes	Yes	Death
21	F	53	Lymphoma	Probable	AmphotericinB	Yes	No	No	No	Death
22	F	60	Lymphoma	Probable	Voriconazole	No	Yes	No	Yes	Unevaluable
23	F	61	CML	Probable	Voriconazole	Yes	No	No	Yes	Unevaluable
24	F	56	AML	Probable	Voriconazole	No	Yes	Yes	Yes	Death
25	F	66	AML	Probable	Voriconazole	No	Yes	No	No	Partial
26	M	56	AML	Probable	Posaconazole	No	Yes	No	Yes	Partial
27	F	53	AML	Probable	Voriconazole	No	No	Yes	No	Partial
28	M	60	AML	Probable	Olorofim	Yes	No	Yes	No	Partial
29	M	40	MDS	Proven	Voriconazole	Yes	Yes	No	Yes	Unevaluable
30	M	71	MM	Probable	Voriconazole	No	Yes	No	Yes	Unevaluable
31	M	73	AML	Probable	Voriconazole	No	Yes	No	No	Partial
32	M	61	AML	Probable	Voriconazole	No	Yes	No	Yes	Progression
33	F	64	Lymphoma	Probable	Voriconazole	No	No	No	Yes	Unevaluable

**Table:** Table 2-2 Clinical and microbiological information of the 33 cases included in the primary cohort and the 20 cases included in the validation cohort PART 2.

Validation cohort										
Case	Sex	Age	Underlying disease	EORTC certainty	Treatment	Previous allo-HCT	Neutropenia	High dose steroids	T-lymphocyte inhibitors	EORTC response week 6
2-1	M	39	MDS	Probable	Posaconazole	Yes	Yes	Yes	No	Stable
2-2	F	66	AML	Prove	Voriconazole	No	Yes	No	No	Partial
2-3	F	34	AML	Probable	AmphotericinB	No	Yes	No	No	Death
2-4	M	34	ALL	Probable	Voriconazole	No	Yes	Yes	No	Stable
2-5	F	66	ALL	Probable	Voriconazole	No	Yes	Yes	Yes	Unevaluable
2-6	F	47	AML	Probable	Voriconazole	Yes	Yes	No	No	Partial
2-7	M	62	AML	Probable	Voriconazole	Yes	Yes	Yes	No	Partial
2-8	F	76	AML	Probable	Voriconazole	No	Yes	No	Yes	Unevaluable
2-9	M	76	Other	Probable	AmphotericinB	No	Yes	No	No	Complete
2-10	M	55	ALL	Probable	Posaconazole	No	Yes	Yes	Yes	Unevaluable
2-11	F	69	AML	Proven	Olorofim	No	Yes	No	Yes	Partial
2-12	M	27	Other	Prove	Isavuconazole	No	Yes	Yes	No	Death
2-13	M	69	MDS	Probable	Isavuconazole	No	No	Yes	No	Partial
2-14	M	29	AML	Probable	Voriconazole	No	Yes	No	No	Partial
2-15	F	44	Other	Probable	Voriconazole	Yes	No	Yes	No	Partial
2-16	F	23	Other	Probable	Voriconazole (+/- SCY-078)	Yes	Yes	No	No	Complete
2-17	M	58	ALL	Probable	Fosmanogepix	No	Yes	No	No	Death
2-18	M	70	MDS	Probable	Voriconazole (+/- SCY-078)	No	Yes	No	No	Complete
2-19	F	70	AML	Probable	Voriconazole (+/- SCY-078)	No	Yes	Yes	No	Complete
2-20	M	71	Other	Probable	Posaconazole	Yes	No	Yes	No	Unevaluable

